# Protecting evolution: How to prepare for attacks against the teaching of evolution

**DOI:** 10.1038/s44319-026-00738-z

**Published:** 2026-03-21

**Authors:** Joel I Cohen

**Affiliations:** Visiting Scholar, Nicholas School of the Environment, Duke University, Durham, NC 277708 USA

**Keywords:** Evolution & Ecology, History & Philosophy of Science

## Abstract

Educators in the life sciences can be challenged by students who disagree with Darwin’s theory of evolution. This article analyses the history of Creationist’s attempts to introduce alternative explanations to Darwinian evolution and makes recommendations on how to prepare for such situations.

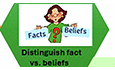

One hundred and one years have passed since the famous Tennessee Scopes Trial concluded in 1925. John T. Scopes, a high-school teacher in Dayton, TN, was accused of violating the Butler Act, which prohibited the teaching of Darwinian evolution in Tennessee schools. Although he was initially found guilty and issued a fine of US$100, his verdict was later overturned by the Tennessee Supreme Court on a legal technicality. What made the Scopes Trial a lasting legacy for teaching education in the USA though was not the outcome but the scathing attacks on Biblical views by Scopes’ lawyer Clarence Darrow and the nation-wide press coverage it received—it was in fact the first trial broadcast on radio.

In the aftermath of the Scopes trial, various states had to revoke laws that would have banned teaching evolution in science curricula. The Butler Act itself was finally repealed in 1967, lasting more than 40 years. Since US courts have had to prevent repeated attempts to include religious interpretations of evolution in science curricula. However, these issues are not settled. The veracity of Darwin’s theory of evolution and its inclusion in science curricula continue to be challenged by religious fundamentalists and not just in the USA.

“The veracity of Darwin’s theory of evolution and its inclusion in science curricula continue to be challenged by religious fundamentalists and not just in the USA.”

Opponents of Charles Darwin’s theory of evolution have used various means to present their opposition (Fig. 1 and Table 1). In addition to such challenges from the outside, some students come to science classes believing in a divinity-based origin of life and the divine creation of humans. Biology teachers will be instructing students raised in fundamentalist households, who have trouble accepting the fact that Darwinian evolution conflicts with the Biblical Genesis and thereby excludes any divine intervention. This article analyses the legal and political history of religious challenges to the teaching of evolution and gives advice to high-school and university teachers faced with students who challenge the teaching of evolution in the classroom.

**Figure 1:**
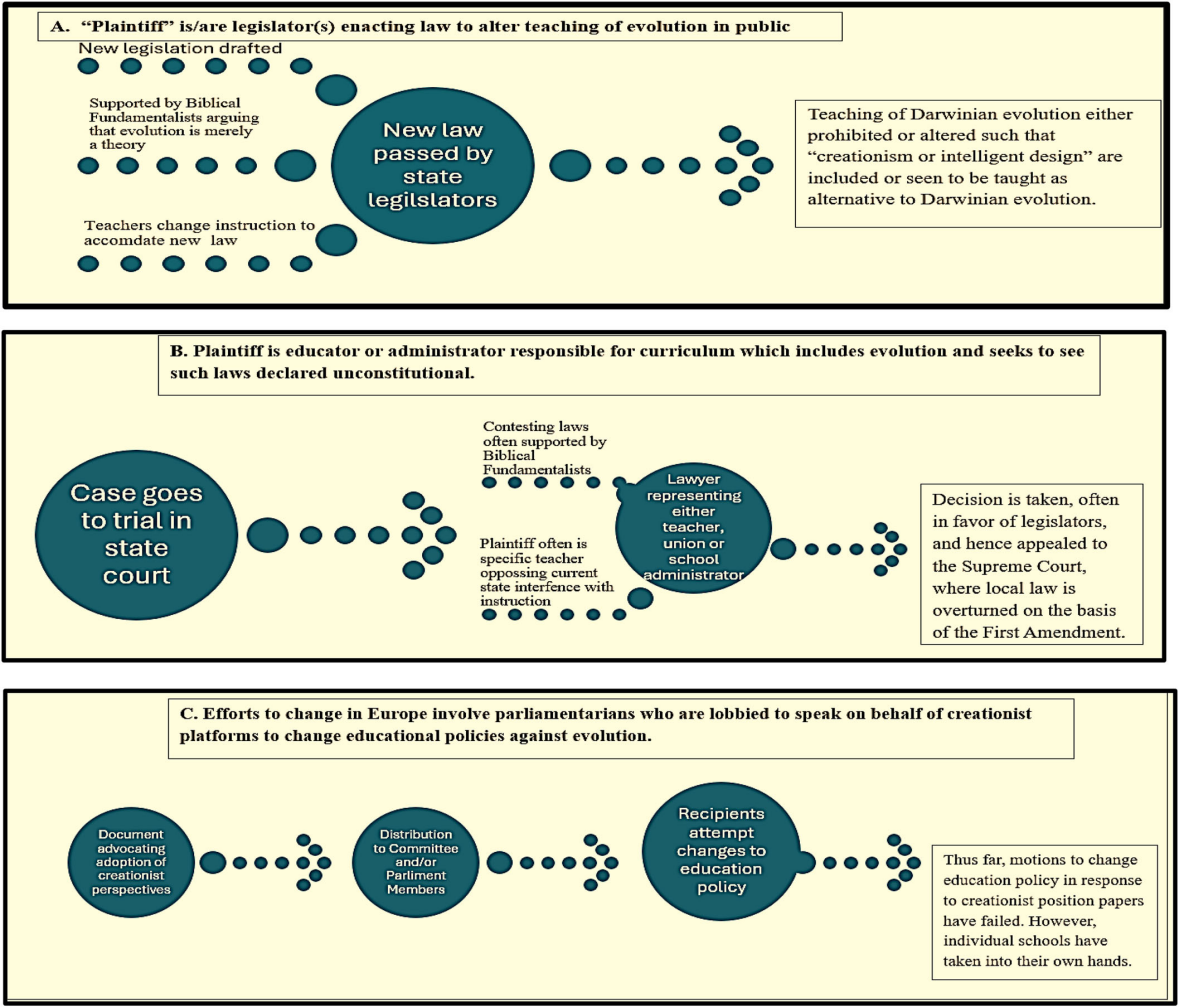
Three approaches for introducing fundamentalist religion-based lessons into public biology and evolutionary education through either legislative, judiciary or parliamentary adjudication. **A** Plaintiff is or are legislator(s) seeking laws which allow fundamentalist approaches to teaching evolution (top panel). **B** Plaintiff is an educator or administrator responsible for setting science curriculum who is seeking curricular changes to allow fundamentalist approaches for evolution (center panel). **C** In Europe, may involve parliamentarians speaking on behalf of fundamentalist arguments for prohibiting the teaching of evolution (bottom panel).

**Table 1:** Key state/federal law(s), cases, and guiding policies regarding the teaching of evolution.

Item#	Year	Location and document or record	Evolution proponent	Anti-evolution proponent	Pertinent laws, legal foundation, and outcomes	Primary tact used in legal defense, either pro/con evolution
1	1789–1791	Constitutional Amendment	--------------	-------------	First Amendment Ratified to law, 1791	Separation of church and state mandated by the Establishment Clause, First Amendment
2	1859	**London:** Publication and reaction to the first edition publication	Charles Darwin	Robert FitzRoy; Richard Owen	Publication of the first edition of *On the Origin of Species by Means of Natural Selection*, or the *Preservation of Favored Races in the Struggle for Life*	-----------------------------
3	1860	**Oxford University**, First oratorial debate; clergy and lay person	Thomas Henry Huxley; Joseph Hooker	Bishop Samuel Wilberforce and others	First confrontation, held during the British Association for the Advancement of Science, Oxford University, possible origins of humankind and *The Origin of Species*	First time for public resistance to the church’s authority regarding human origins
4	1866–1868	Constitutional Amendment	-------------------------	---------------	Fourteenth Amendment Ratified to law, 1868	The law’s due process protection prevents the government from promoting religion
5	1925	**Tennessee:** Law prohibit**s** teaching of evolution	-----------------------	Legislators	The Butler Act, passed in state of Tennessee. Unlawful to teach any theory which denied man’s divine creation.	Prohibited teaching of the Evolution theory in all Universities, Normals, and other Public Schools
6	1925	**Tennessee:** Legal court case, against the teaching of evolution	John Scopes; Clarence Darrow	William Jennings Bryan	Scopes charged with violating Butler Act. Religious arguments dismissed; would only allow discussion of alleged Butler Act violation.	Scopes violated the Butler Act; Intelligent Design
7	1968	**Tennessee:** Butler Act finally removed	-----------------------	Tennessee school system	**Supreme Court ruled Butler Act unconstitutional**.	Supreme Court relied on use of First Amendment
8	1968	**Arkansas:** Required several court decisions until the final.	Susan Smith Epperson --public school biology teacher (plaintiff)	State of Arkansas	Epperson V. State of Arkansas	Susan Epperson went before Chancery court. Chancery court ruled statue violated 14^th^ amendment.
9	1981	**Arkansas** Act that became law in March 1981.	Bill McLean; ACLU organized those opposing the law	Biblical Fundamentalist	State of Arkansas, Act/bill 590, Balanced *Treatment for Creation-Science and Evolution-Science Act*: equal class time on “creation science” as for Darwinian evolution.	Trial set to determine if such was legal: District Court, Little Rock, AR: Injunction entered permanently prohibiting enforcement of Act 590.
10	1981	**Louisiana:** State Act	Don Aguillard, Science & Assistant Principal	Louisiana State Act	“Balanced Treatment for Creation-Science and Evolution-Science in Public School Instruction” Act	Don Aguillard charged that it was in violation of the establishment clause
11	1987	**Louisiana**: First Amendment -defeated **local** law	Don Aguillard	State legislators	**Supreme Court struck down Louisiana creation law**, ending effort to teach students creation science if they learned about evolution	Edwards v. Aguillard, June 19, 1987. The law was really to promote religion.
12	1999	Science and Creationism: A View from the National Academy of Sciences	National Academies of Science (NAS)	-------------------------	---------------------------------------------	-----------------------------------------------------
13	2004	**Wisconsin** – state-mandated teaching evolution	------------------------	City School Board determined: teach pros/cons of evolution	Grantsburg became the first school board/district to allow teaching various origins of life theories during evolution. Teaching intelligent design was not mandated.	Guidelines revised, 2004, requiring students be able to articulate the strengths and weaknesses of evolutionary theory
14	2005	**Pennsylvania**: requirement defeated; found unconstitutional	Tammy Kitzmiller, Glenn Branch – (*National Center for Science Education*)	Dover School District	Kitzmiller v. Dover, Pennsylvania, Tammy Kitzmiller, Plaintiff, v. Dover area school district, Defendants.	Teachers required to recommend to students “intelligent design,” as alternative to evolution. Unofficially ended ID claims
15	2006	Resource for teachers, legislators, and policymakers	AAAS, Board of Directors	--------------	Statement on the Teaching of Evolution	“There is no significant controversy surrounding Evolution within the scientific community about the validity of the theory of evolution.”
16	2007	Summary paper	Pew Research Center Report (Masci 2007)	--------------	Twenty years after a landmark Supreme Cout Decision, Americans are still fighting about evolution	A detailed study conducted in 2006 on the acceptance of evolution vs. other options
17	2007	**Kansas:** Decision by School Board	Public School Science Teachers	Intelligent Design Network	On 2/13/2007, the Kansas Board of Education tossed out Creationist science standards	“The new standards will help to strengthen science education in Kansas.”
18	2012	**Tennessee** Law repealed in 2017 with new bill passed and signed by Gov Haslam	-----------------	Discovery Institute, a proponent of intelligent design	Legislators in Tennessee approved a law obligating teachers to “present scientific strengths and scientific weaknesses of existing scientific theories”	No definition provided for strengths and weakness and not signed by the Governor.
19	2016	Creationism and the Law	NCSE (National Center for Science Education)	---------------	Website showing the legal history of creationist cases against evolution, coupled with many other types of information	------------------------------------------------------
20	2019	Position on Teaching Evolution	National Association of Biology Teachers	-------------------	Adjusted in 2019 to consider new developments in textbooks and advocacy	
21	2022	Website hosted & designed by Museum of Paleontology	NABT	--------------	Understanding Evolution – website, University of California, Museum of Paleontology, Berkeley	NABT members, teachers, and administration
22	2023	**Montana**: State legislative Senate Bill 235	--------------	Republican Senator Daniel Emrich from Great Falls	Bill introduced in Montana prohibits instruction on matters that are not scientific fact	Another state “attacking scientific theories.” Upon review, the bill was tabled and has not moved forward.
23	2023	**Oklahoma:** SB140- Science Education Act	-----------------	Republican Senator Nathan Dahm	Bill to “understand, and review in an objective manner scientific strengths and weaknesses of existing theories”	Bill died in the Senate Education Committee on March 23 when the deadline for action passed.
24	2023	Position description without evolution	NSTA	---------	National Science Teacher Association – Life Science Teacher Job Description Template	Evolution **not** listed as key concept in list for curricular preparation
25	2024	**West Virginia:** Bill into Law despite lawsuit concerns	------------------	Amy Grady (R- District 4)	West Virgina; new law allows questions from students about scientific theories	Bill protects teachers discussing intelligent design
26	2025	Background; title should be states allow evolution & ID to be taught.	---------------	States that Don't Teach Evolution 2026 https://worldpopulationreview.com/state-rankings/evolution-teaching-states	URL showing state by state status as to offering instruction in creation science along with Darwinian evolution	Survey: found 6 states required if evolution is taught, then so too creationism
27	2025	**North Dakota:** State legislative bill. Senate Bill 2355	Opposing members of the state senate education committee	Sen. M. Dwyer, R-Bismarck, State Senate Education Committee proposing bill	North Dakota bill would have required inclusion of “intelligent design” in state science standards and see Intelligent Design as a scientific theory.	NCSE (National Center for Science Education). Bill was defeated by vote of the Education Committee, 25–22.

## Charles Darwin and the first debate on evolution

An apt response to religious concerns could surely include comments by Darwin himself: “I can see no limit to the amount of change, to the beauty and infinite complexity of the coadaptations between all organic beings, one with another and with their physical conditions of life, which may be effected in the long course of time by nature’s power of selection” (Darwin, 1859). He concluded: “When I view all things not as special creations, but as the lineal descendants of some few beings which lived long before the …Silurian…was deposited, they seem to me to become ennobled.” This effectively removed divinity from the emergence of life and raised the ire of those believing in Genesis to explain how life came about.

Not surprisingly, the first debate pitting Darwin’s version of evolution against a literal interpretation of the Bible occurred some 65 years before the Scopes trial between Bishop Samuel Wilberforce and Thomas Henry Huxley, acting as a stand-in for Charles Darwin. It was held during a meeting of the British Association for the Advancement of Science at Oxford University in a session designed to promote discussion of *On the Origin of Species’* just 8 months after its publication in November 1859. Even with such a brief time to read and review, significant opposition to Darwin’s book had already developed.

Beyond the debate between Wilberforce and Huxley, Darwin, his grand theory, and the implicit notion that man himself has descended from apes became the target of much ridicule and scathing attacks in the press. The immediate rejection of his theory of how species change over time and the attacks on them were not surprising to Darwin. However, the magnitude of such dissent was, as it was coming from many of his previous professors, staff at the Museum of Natural History in London, and even those who served with him aboard the *HMS Beagle*. Gradually, this dissent would subside as there were many personal reasons that people rejected his book (Karp, 1968).

“… Darwin, his grand theory, and the implicit notion that man himself has descended from apes became the target of much ridicule and scathing attacks in the press.”

Darwin had prepared for this type of response by asking colleagues to read parts of his draft. Despite harboring concerns by family and peers, he proceeded with his publisher, John Murray, until *On the Origin of Species* was eventually printed some 23 years after his voyage aboard the *HMS Beagle*. In fact, he was able to see edition after edition of the *Origin* off to the printers thanks to his fortitude and persistence (Cohen, 2016).

## Why prepare now?

One might expect that, after Bishop Wilberforce’s arguments were countered by Thomas Huxley, Clarence Darrow countered William Jennings Bryan and the revocation of the Butler Act, Darwinian evolution would be left to stand the test of time. In 1996, even Pope John Paul II pronounced that “organic evolution could not be dismissed as pure theory” (Mislin, 2025). Other clergy have also voiced their support for the teaching of evolution, and the desire to see these teachings reconciled with religion (Zimmerman and Hunt, 2025).

On the other side, scientists, educators, and scientific organizations, supported by courts, have upheld a clear separation of scientific theory and religious beliefs in education. As the US National Academies of Science (NAS, 1999) stressed: “While the mechanisms of evolution are still under investigation, scientists universally accept that the cosmos, our planet, and life evolved and continue to evolve. Yet the teaching of evolution to schoolchildren is still contentious. In *Science and Creationism*, The National Academy of Sciences states unequivocally that creationism has no place in any science curriculum at any level.”

But rather than seeing attacks on evolution subside, just the opposite occurred. As evolution became more accepted and an integral part of scientific education, concerns from Biblical Fundamentalists increased, worried that if evolution went unchecked, its impact may go well beyond biology by affecting broader social values. Consequently, their arguments moved from a literal interpretation of the Bible to “Intelligent Design”, and they persuaded politicians, school boards, and parents to include this perspective into science curricula. Indeed, some US states and European schools have allowed such combined studies of Creation or Intelligent Design and Darwinian evolution—and were subsequently and successfully challenged by scientists and teachers (Table 1).

“As evolution became more accepted and an integral part of scientific education, concerns from Biblical Fundamentalists increased, worried that if evolution went unchecked, its impact may go well beyond biology…”

However, just as important as knowing what happened, when it happened, and how such legislation was implemented, is knowing *why* these events occurred. Indeed, there are some commonalities among the various challenges to the teaching of evolution (Table 2). It is the why, or the reasons behind these commonalities, which are based on a supposed denial of divinity. Religious concerns, then and now, regarding public education come from a literal interpretation of the Bible and Creation. Hence the name, Biblical Fundamentalists (Fowler, 1999-2000), to describe individuals who believe that science instruction of evolution must reflect a divine presence. In addition to science curricula, the insertion of religion includes efforts to give public schools and state colleges the right to employ chaplains or to mandate the display of the Ten Commandments.

**Table 2:** Common issues arising from the adjudication of either legislative or policy initiatives (Table 1) that limit or curtail the teaching of evolution by increasing the emphasis on non-scientific alternatives.

1. Those directly involved in teaching evolution are as susceptible as their curriculum to the pressures of adding non-scientific alternatives for change over time in biological organisms.	2. In such instances, the teacher can become either plaintiff or defendant.
3. School administrators and teachers have allowed religiously motivated “alternatives” to evolution to appear in textbooks and in science curricula.	4. Debates over the meaning of the word ‘theory’ are being used to create confusion and doubt over the theory of evolution to argue for equal time for Biblical interpretations of origin stories.
5. Amendments and other legislation that ensure the separation of state and religion have been effective thus far in curtailing religiously motivated interventions in education.	6. Most religious interventions arise due to the perceived lack of divinity in the origin of the human species and the equivalence of *Homo sapiens* with other species on the tree of life.

After the Butler Act and similar laws were repealed in the late 1960s, the fundamentalist movement garnered renewed momentum with the advent of the Moral Majority, beginning in 1979 as a conservative political party (Mislin, 2025). As voiced by Fowler (1999-2000): “Darwinian evolution has become one of the pivotal intellectual issues of our time because it influences so many of the ways in which we organize our experience and ground our beliefs.” The movement also evolved its arguments: instead of outright denying Darwin’s theory of evolution and trying to introduce a literal interpretation of the genesis into school curricula, they questioned the meaning of the word “theory” confusing it with its more common meaning, and renamed “creationism” to “intelligent design” so as to semantically downplay religious tones. Here, the use of the common meaning would deliberately confound what is meant by an assumption, often based on limited or no actual information, as opposed to its meaning in science: a set of principles capable of explaining a wide set of facts, having stood the test of time and expert review.

The most recent case for adjudication occurred in North Dakota, where the Education Committee defeated Senate Bill No. 2355, which would have required state science standards to include intelligent design. From the time of the Scopes trial to the North Dakota bill, 11 other cases were resolved (Table 1) with two by the US Supreme Court, one by a federal court, and nine by state legislatures, school boards, or state courts.

Other parts of the world have also seen attempts by religious fundamentalists to attack the teaching of evolution. When the Culture Committee in the Council of Europe issued a report that creationism’s messages should not be considered scientific, it was immediately followed by pro-Creationist statements in protest from the Netherlands, Poland, Serbia, and Italy (Gross, 2002). Politicians in Europe have not only condoned creationist petitions but actually supported them (Blancke et al, 2013). The UK Prime Minister at the time, Tony Blair, refused to criticize the teaching of alternative views of evolution after a public school in the UK was found presenting creationist views as a respectable alternative to the school’s biology textbook (Gross, 2002).

In Turkey, creationists are represented in several governmental parties in parliament, which makes it difficult for teachers to oppose the distribution of creationist books in schools (Koenig, 2001). In Israel, a chief scientist in the education ministry called both evolution and global warming unreliable and for curricula to go through religious censorship to cast out evolution. Even while most of the press coverage focuses on the USA, many other countries are embroiled in similar disputes with religious fundamentalists.

“Even while most of the press coverage focuses on the USA, many other countries are embroiled in similar disputes with religious fundamentalists.”

As seen, the attempts to influence the teaching of evolution differ between countries with a national educational policy or ministry for education and the state-based situation of the USA. Consequently, religious fundamentalists in the USA mostly target state legislators or local school boards and are resolved by legislative or legal actions, whereas fundamentalists elsewhere seek to influence educational policies through bureaucratic means. The following analysis of historically important cases and the subsequent recommendations for dealing with religious objections to the teaching of evolution—while largely focused on the USA—are just as relevant to other countries as well.

## Learning from history in preparation for the challenge ahead

The study of cases in which religious fundamentalists tried to challenge the teaching of evolution can also help prepare educators for such future encounters. While the Scopes case, when compared to current legal battles, received the greatest notoriety, such debates and legal arguments continue to occur as Biblical Fundamentalists (Fowler, 1999-2000) attempt to insert their own religion-based scientific interpretations in school curricula. From the cases listed in Table 1, four key objections against the teaching of evolution can be identified.

One primary objection against teaching evolution—and to Darwin’s theory itself— emerged directly after publication of *On the Origin of Species*. It takes issue with the placement of the human species, as Darwin pictured it, as just another branch on the tree of life, equivalent to other species.

A second objection is the belief that each human soul is created by a divine being, which cannot be explained through natural causes (Mislin, 2025).

A third objection is the discrepancy between the geological age of the Earth and the biblical account of creation, which has inspired creationists to align geological evidence with the scriptures (Wise, 1998).

Finally, there are attempts to integrate scientific knowledge into religious worldviews, referred to as “integrated education” (Coche and Jansen, 2025), which gave rise to ‘intelligent design’ as an alternative to the theory of evolution.

These four arguments are primarily religious in nature and therefore are prevented from being imposed on public educational institutions by the Constitutional Amendments 1 and 14 in the USA. The First Amendment mandates the separation of church and state, while the 14th Amendment mandates the right to due process, which means that any attempts to change science curricula can be challenged in courts (Table 1, items 1 and 4).

Another notable aspect is the timeline of legal challenges. In the 100 years since the Scopes trial, only two other court cases appeared in court during the first 50 years until 1975, but ten more cases were filed between 1975 and 2025. This increase occurred even though many of the earlier cases were dismissed by the State Supreme Courts— Tennessee, Arkansas, and Louisiana—or local, district, or state courts (Table 1). This lack of initial success from the fundamentalists’ perspective inspired another approach: creating confusion around the meaning of the word “theory” as an argument in favor of teaching alternative explanations. However, the courts have been siding with public school boards, declaring petitions for alternative instruction of non-Darwinian evolution unconstitutional.

“In the 100 years since the Scopes trial, only two other court cases appeared in court during the first 50 years until 1975 but ten more cases were filed between 1975 to 2025.”

Table 1 also includes statements about evolution and creationism from key scientific organizations that explain and clarify policies for education purposes, including the AAAS, the NAS and the National Association of Biology Teachers. The latter calls on teachers to reject “… calls to account for the history of life or describe the mechanisms of evolution by invoking any non-natural or supernatural notions, whether under the banner of “scientific creationism,” “intelligent design,” or similar designations.”

Although all cases in Table 1 have been settled, more may come. Will professionals in the life sciences and in education be prepared to respond? Even though 166 years have passed since the encounter between Bishop Wilberforce and Thomas Huxley, and 100 years since the trial of John T. Scopes, challenges by conservative religious groups continue even as more evidence supporting the theory of evolution has become available for teaching (Greenwood, 2013). But this evidence has had no influence on those who adhere to the “Divine creation of man.” Their beliefs stem from a literal interpretation of the Biblical Genesis, such that “God made the earth and all plants and animals in six days, and major geological features appeared after a Great Flood. Evolution is a fallacy; humans were created in their present form by divine fiat 6000 years ago” (Milner, 2009).

## Prepare for challenges

Evolution can be a difficult unit to teach, even without having to prepare for counterarguments to religious fundamentalism. As if that were not enough, teachers, such as John Scopes, can be targeted along with their curriculum to accommodate intelligent design or creationism. This approach puts the teachers at risk of becoming a plaintiff or a defendant. Other teachers, either by school rule or law, have allowed religiously motivated “alternatives” to appear in a science curriculum. These often come under the disguise of “intelligent design” or “creation science. Language that supports such ‘alternative’ concepts has already appeared in some science textbooks, supported by Biblical Fundamentalists. Thus, timely preparation by educators becomes all the more important.

**Figure 2:**
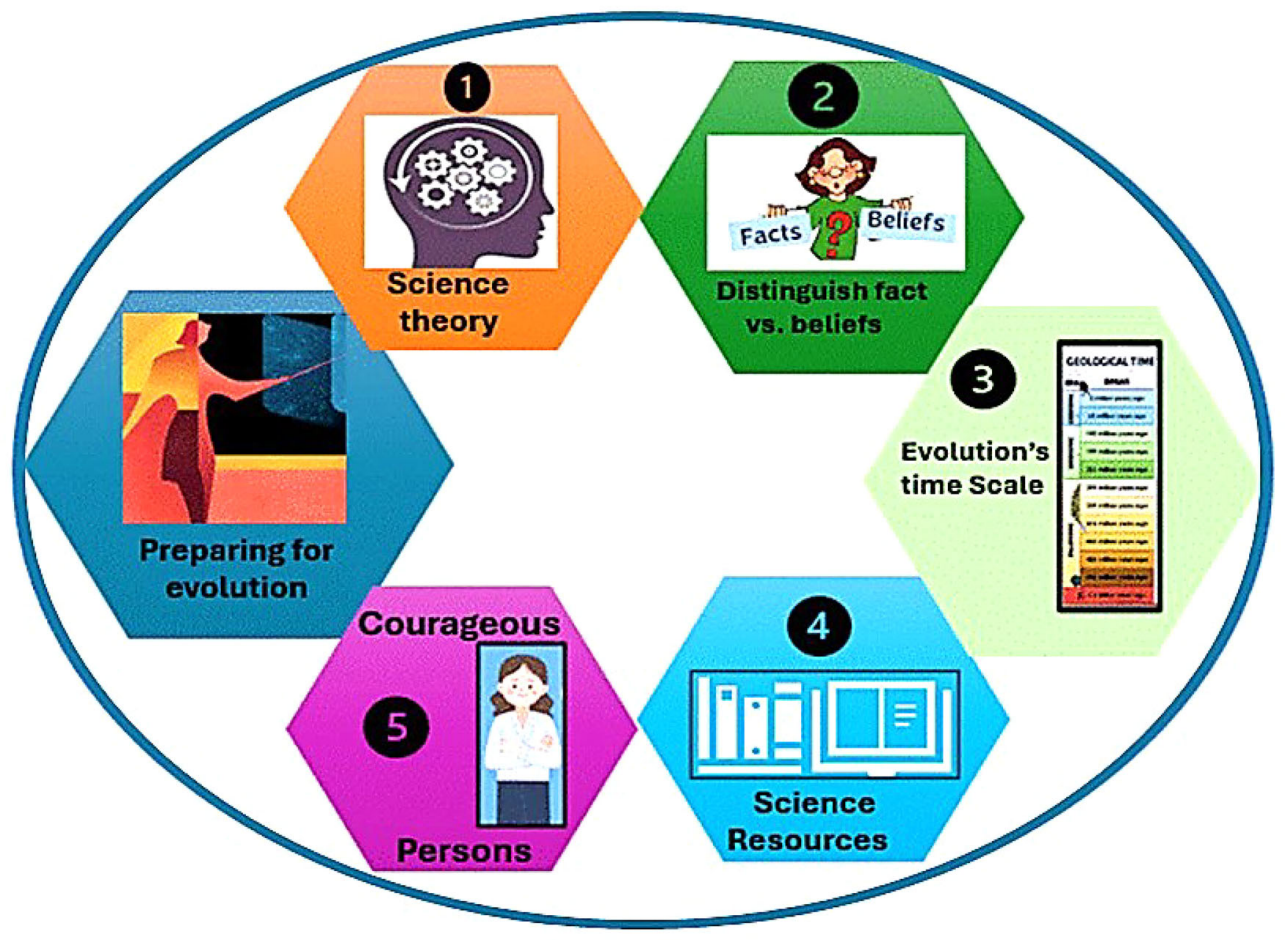
Five topics to study in preparation for claims by biblical fundamentalists that counter Darwinian evolution. The first icon regards the use and meaning of the word “theory”. The second represents separating evolution facts from students’ beliefs.” The third icon is to ensure understanding of the biblical versus geological time taken to produce life on earth. Fourth is the icon for reading literature regarding evolution from scientific societies and from religious organizations. The fifth focuses on courageous individuals, both contemporary and in the past, who adhered to the practice and teaching of evolution as defined by science.

Figure 2 illustrates five topics that educators can use to counter claims by biblical fundamentalists. Educators benefit from these topics when they are confident of their distinctions and meaning. which comes from mastering each one prior to meeting students. The first topic addresses the use of the word “theory”. The lack of initial success from the fundamentalists’ perspective has inspired creating confusion around the meaning of the word “theory” as an argument in favor of teaching alternative explanations. Educators should be clear in their use of the term, as in scientific theories, which differs from its use by lay people. As we have seen, what began with creationism or creation science has become intelligent design and moved on to focus on the word theory. This is based on the vagueness of the word theory itself, especially in common vernacular to mean “guess” (NRC, 2012) as opposed to its specific meaning in science, where it describes a tested and validated hypothesis that is accepted as true unless proven otherwise.

As defined in the Next Generation Science Standards (NGSS, 2013), theories are “based on significant bodies of knowledge and evidence, are revised in light of new evidence, and withstand scrutiny by the scientific community.” This definition clearly separates its scientific meaning from that of a guess, which otherwise creates a sense of confusion among state school boards and politicians to believe incorporating intelligent design into a unit on evolution clarifies its meaning.

Second is the ability to separate fact from beliefs, including personal beliefs from established facts based on testable and validated theories. Clarifying the difference early in teaching science lays the groundwork and can be refreshed as needed and relevant to a given lesson. In addition, it helps educators to reject attempts “to account for the history of life or describe evolution by invoking any non-natural or supernatural notions”.

Third comes the geological time scale versus Bible-based earth history, which helps to counter Biblical interpretations of the age of the Earth and a divine creation of humans (Wise, 1998). This icon represents the important distinctions between geological eras and biblical time calculations for those who adhere to the “Divine creation of man.” Their beliefs stem from a literal interpretation of the Biblical Genesis, such that “God made the earth and all plants and animals in 6 days, and major geological features appeared after a Great Flood. Evolution is a fallacy; humans were created in their present form by divine fiat 6000 years ago” (Milner, 2009).

Thus, it is important to cover the history of Earth from its formation around 4.5 billion years ago (BYA), with known fossil-based life showing eukaryotes at 2.1 BYA and multicelled life not appearing until 700 MYA, followed by the Cambrian explosion at 535 MYA, and eventually the emergence of land tetrapods at 365 MYA. Emphasis should be placed on the great amount of time documented for these major changes to occur, often requiring millions of years to explain that evolution works over long timescales, much more extensive than that expressed in Biblical time.

The fourth step in preparation is to utilize and understand statements concerning evolution and creationism from key scientific organizations. These reports clarify policies for education purposes, including the AAAS, the NAS (1999), and the National Association of Biology Teachers. In addition, following the strategies and arguments of creationists can also be helpful in preparing answers and counterarguments for teaching evolution. Some sources to this end include “Answers in Genesis Canada;” *Kansas v. Darwin*, a 2008 documentary by Unconditional Films; or the creationist Discovery Institute, for example.

The final suggestion is to recognize courageous individuals who have challenged attempts to instill Creationist material into curricula or censure instructors who teach evolution. Examples of notable individuals include Susan Epperson (Item 8); Don Aguillard (Items 10 and 11); Tammy Kitzmiller (Item 13) and Bill McLean (Item 9). In addition, lessons could also include scientists’ biographies focusing on obstacles faced in the advancement of their work or theories. This allows students to identify with such personal struggles (Clough, 2024). Accommodating such biographies was the subject of a recent Gordon Cain conference (SHI, 2024), including a study on introducing women naturalists in the biodiversity section of an evolution unit (Cohen, 2024). This could also allow for a similar biographical lesson on Darwin, highlighting his own personal struggles to publish *On the Origin of Species* (Cohen, 2016).

## Closing thoughts and concerns

While some suggest that “teaching evolution has a bright future in the U.S.”, this author finds many indications to the contrary given the current political environment. What then can be done to prepare for future encounters with religiously motivated students? The following points offer preemptive steps for educators to consider before teaching evolution and a way ahead should such situations arise.

The separation of religious beliefs and scientific facts in the classroom—upheld by courts and policymakers during the past 100 years—should not be taken for granted. Federal funding for research and education has already been cut in the current political environment and is still in jeopardy. Should opinions change at the judicial level on the natural selection-to-intelligent design continuum by accepting intelligent design as a viable alternative to the theory of evolution, then funding for evolution-related research and education could be further cut.

“The separation of religious beliefs and scientific facts in the classroom – upheld by courts and policymakers during the past 100 years – should not be taken for granted.”

One thing is certain since the Scopes trial: all sides in the debate have improved their messaging, lobbying, and political posturing, and there are far more actors than in the past. Thus, the more prepared evolutionary biologists are when it comes to future attacks, the better. This may mean looking elsewhere for funding, as well as being ready and able to contribute to counter intelligent design challenges.

“One thing is certain since the Scopes trial: all sides in the debate have improved their messaging, lobbying and political posturing, and there are far more actors than in the past.”

One can but wonder why at this time is there such a concern regarding the teaching of evolution. An answer to this question would help bridge the gap and mend fences. Until then, the words of Tammy Kitzmiller, the lead plaintiff in the Dover, Pennsylvania case (Table 1), are as accurate now as when she said them in 2005: “People need to keep an eye on this. They need to keep their science classes straight and need to keep the school focused on science in the science classroom.”

## Supplementary information

Peer Review File
