# Supplementary material for: Protecting evolution: How to prepare for attacks against the teaching of evolution
Source: EMBO Rep. 2026 Mar 21;27(8):1861–9. doi: 10.1038/s44319-026-00738-z (PMC13121594; doi:10.1038/s44319-026-00738-z)
Supplement: Supplementary file 1 — Peer Review File [file 44319_2026_738_MOESM1_ESM.pdf]

EMBO reports

Protecting Evolution: How to prepare for attacks against the teaching of evolution

Joel Cohen

Corresponding author(s): Joel Cohen (joel.cohen@duke.edu)

Review Timeline:

Submission Date:	1st Dec 25
Editorial Decision:	9th Dec 25
Revision Received:	19th Jan 26
Accepted:	24th Feb 26

Editor: Holger Breithaupt

Transaction Report: This article was editorially reviewed.
